# Changing Effects of Minimally Invasive Surgical Intervention on ALT, AST, and UA in Patients with Obstructive Sleep Apnea-Hypopnea Syndrome

**DOI:** 10.1155/2022/3622896

**Published:** 2022-07-14

**Authors:** Liang-Cai Yu, Xin Zhao, Li-Jun Liu, Xiao-Ying Li, Jin Zhou, Ping Zeng, Xiao-Qing Zhang

**Affiliations:** ^1^Department of Occupational Health, Sleep Medicine Center and ENT, West China School of Public Health and West China Forth Hospital, Sichuan University, Chengdu 610041, Sichuan, China; ^2^Department of Administration, West China Second University Hospital, Sichuan University, Chengdu 610041, Sichuan, China

## Abstract

**Background:**

This study aims at exploring the effect of obstructive sleep apnea–hypopnea syndrome (OSAHS) on the liver and kidney function indexes of patients and analyze the changes in these indexes after minimally invasive surgery.

**Method:**

Patients with OSAHS (*n* = 51) who were diagnosed via polysomnography (PSG) and received minimally invasive surgery in the sleep disorders diagnosis and treatment center of the West China Fourth Hospital of Sichuan University from January 2017 to January 2019 were selected as test subjects and placed in the OSAHS group. At the same time, 79 healthy people with no snoring or breathing difficulties were selected from the medical examination center of the hospital as the control group (tested as normal by PSG). These two groups were used to compare the differences in the related indexes of serum liver and kidney function and evaluate the changes in sleep monitoring and related liver and kidney function indexes in patients with OSAHS after minimally invasive surgery.

**Results:**

The alanine aminotransferase (ALT), aspartate aminotransferase (AST), and uric acid (UA) levels were higher in the OSAHS group (48.98 ± 36.34, 28.88 ± 14.80, and 422.30 ± 98.65, respectively) than in the control group (21.91 ± 11.61, 22.18 ± 6.19, and 330.49 ± 64.45 and *t* = 6.514, 3.549, and 6.373, respectively; *p* < 0.05). Of the patients with OSAHS, 17 were followed up for one year. After minimally invasive surgery, ALT decreased from 44.29 ± 20.61 to 26.47 ± 9.91 (*t* = 4.395), AST decreased from 27.71 ± 8.32 to 21.82 ± 4.81 (*t* = 3.673), and UA decreased from 397.35 ± 92.14 umol/L to 362.94 ± 106.76 umol/L (*t* = 2.580), and these differences were statistically significant (*p* < 0.05).The changes in ALT (*r* = −0.635) and AST (*r* = −0.504) were related to the difference in the lowest blood oxygen saturation (*p* < 0.05), and the change in UA was related to the difference in the apnea–hypopnea index (*r* = −0.532, *p* < 0.05).

**Conclusion:**

There are some abnormalities in liver- and kidney-function-related indexes in patients with OSAHS, and minimally invasive surgery can help to improve liver and kidney function in these patients.

## 1. Introduction

Obstructive sleep apnea–hypopnea syndrome (OSAHS) refers to an apnea–hypopnea index (AHI) ≥ 5 times/hour in a night consisting of at least seven hours of sleep, and it is a clinically common sleep and respiratory disease with obstructive respiratory events [1]. Patients with OSAHS suffer from chronic intermittent hypoxia due to repeated apnea or sleep apnea at night, increased sympathetic excitability of the body, and increased oxidative stress response, leading to metabolic disorders of the body and causing damage to multiple organs and systems [2]. Previous studies have shown that OSAHS is an independent risk factor for diseases such as hypertension, stroke, and type 2 diabetes [3, 4]. With the deepening of research, increasing numbers of studies have also found a correlation between OSAHS and liver and kidney function damage [5, 6]. In addition, studies have shown that OSAHS is considered to be an independent risk factor for nonalcoholic fatty liver disease (NAFLD) [7, 8]. Krolow GK assessed the severity of OSAHS and NAFLD. The degree of correlation found that regardless of obesity or not, there was a correlation between liver fibrosis and moderate to severe OSA [9]. OSAHS patients are prone to kidney damage due to long-term hypoxemia and hypercapnia. The risk of chronic kidney injury in OSAHS patients is 4.542 times higher than that of the general population. In recent years, minimally invasive surgery has achieved good results in the treatment of OSAHS. Our team has also conducted in-depth research on the treatment of OSAHS by minimally invasive surgery. We believe that minimally invasive surgery is an effective method for the treatment of OSAHS [10–12]. But there have been few reports relating to the changes in the liver and kidney function indexes in patients with OSAHS after minimally invasive surgery. Therefore, this study will further analyze the changes in the liver and kidney function indexes in patients with OSAHS and evaluate the impact of minimally invasive surgery on related liver and kidney function indexes in these patients.

## 2. Methods

### 2.1. Subjects

OSAHS group: a total of 51 patients who came to the Department of Otorhinolaryngology of the West China Fourth Hospital of Sichuan University for snoring and sleep apnea, were diagnosed by polysomnography (PSG) as having OSAHS, and who received minimally invasive surgery from January 2017 to January 2019 were selected as the OSAHS group.

Control group: a total of 79 healthy people with no snoring or sleep apnea symptoms were selected from the physical examination center of the Fourth Hospital of West China as the control group which was diagnosed by PSG (AHI<5).

Exclusion criteria were as follows: (1) having received OSAHS-related treatment before the current treatment, such as surgical, drug, or continuous positive pressure ventilation treatment; (2) suffering from serious cardiovascular, cardiopulmonary, or cerebrovascular diseases; malignant tumors and other organics; sexual diseases; insomnia; or anxiety, depression, or other neuropsychiatric diseases; and (3) having incomplete clinical case data.

This study was conducted with approval from the Ethics Committee of West China School of Public Health and West China Forth Hospital (HXSY-EC-2021022). This study was conducted in accordance with the Declaration of Helsinki. Written informed consent was obtained from all participants. All subjects were informed of the purpose of the study and agreed to their clinical data being used for the study.

### 2.2. Materials

#### 2.2.1. The PSG

In this study, the German SOMNO screen™ plus PSG monitor was used for sleep monitoring for at least seven hours. All study subjects were told in advance not to overwork; take drugs that affect sleep; or drink alcohol, coffee, strong tea, or other beverages on the day of sleep monitoring to ensure normal sleep at night. After the sleep monitoring, data were automatically analysed by the computer and checked by a sleep technician. The data collected were the apnea–hypopnea index (AHI), lowest arterial oxygen saturation (LsaO_2_), and the microarousal index.

### 2.3. Hematological Examination

All patients with OSAHS are required to submit to a hematological examination before undergoing minimally invasive surgery. The patient took fasting venous blood after 8 o'clock on the second day after fasting and used a disposable venous needle to collect blood from the elbow vein and an orange fast serum tube for blood collection and storage. The serum was extracted for inspection of corresponding indicators. We will follow up patients who have undergone surgery for one year, ask them for relevant information, and collect blood. The blood biochemical index of the control group was derived from the physical examination results. The data collected were the alanine aminotransferase (ALT), aspartate aminotransferase (AST), urea, creatinine, uric acid (UA), and other indicators. All the above hematological examinations were conducted by the laboratory department of the West China Fourth Hospital of Sichuan University.

### 2.4. Minimally Invasive Surgery

The diagnostic and treatment center for sleep and respiratory diseases at the Fourth Hospital of West China developed a minimally invasive surgery system by combining the American Ellman radiofrequency knife and the low-temperature plasma system [10], which has been used in the treatment of patients with OSAHS for many years. Local anesthesia is used for this operation, and we choose different surgical methods according to the different conditions of the patient. We use povidone-iodine solution to disinfect the surgical site before the operation. 1% tetracaine was used for topical anesthesia, and 1% lidocaine was used for local infiltration anesthesia. Then the turbinates, soft palate, uvula, tonsils, and tongue base were operated on.

1. Inferior turbinate: by using the low-temperature plasma system (Reflex45 cutter head), surgical electrodes are inserted into the middle and lower portions of the front edge of the inferior turbinate in multiple levels, and advanced from front to back until they are close to the back of the inferior turbinate, and they ablate for about 10–15 seconds.
2. Oropharyngeal plane: ablate the soft palate, uvula, and tonsils, and make an outward 45° “V”-shaped incision along the junction of the soft palate and the uvula on both sides with a radio frequency electric knife (U-shaped blade) to the tongue and palate arch and remove the surrounding hypertrophic palate tissue. For the thick uvula, part of the uvula can be cut off.
3. Tongue plane: A low-temperature plasma system (Reflex55 cutter head) was used, which was generally inserted in and around the midline of the tongue base. 4 or 6 treatment points (1.0∼1.5 cm apart) were selected to insert electrodes into the mucosa, and then they were moved backward and downward for about 1.0 cm, lasting for about 10 s.

The patients were observed in the postoperative ward for 5–7 days, and intravenous antibiotics were used to prevent infection and were supplemented with local semiconductor laser irradiation treatment for inflammation and swelling.

### 2.5. Data Processing and Statistical Analysis

The EpiData 3.3 software was used to input the data, and SPSS 22.0 was used for statistical analysis. Quantitative data were expressed as mean ± standard deviation, and count and grade data were expressed as a frequency or composition ratio. The independent group *t*-test was used for comparison between groups, and the paired *t*-test was used for comparison before and after minimally invasive surgery. The correlation analysis for continuous variables was done using Pearson correlation analysis. *p* < 0.05 was considered to be statistically significant.

## 3. Results

### 3.1. Comparison between the OSAHS and Control Group

As shown in Table 1, there was no statistically significant difference between the groups in terms of gender, age, occupation type, smoking habits, or alcohol consumption habits (*p* > 0.05).

As shown in Table 2, it was found that the levels of ALT, AST, and UA in the OSAHS group (48.98 ± 36.34, 28.88 ± 14.80, and 422.30 ± 98.65, respectively) were higher than in the control group (21.91 ± 11.61, 22.18 ± 6.19, and 330.49 ± 64.45 and *t* = 6.514, 3.549, and 6.373, respectively; *p* < 0.05). This suggests that patients with OSAHS may have abnormal liver and kidney metabolism.

### 3.2. Changes in the Clinical Indexes of Patients after Minimally Invasive Surgery

In this study, a total of 17 patients with OSAHS were followed up after minimally invasive surgery. As shown in Table 3, their clinical indicators were compared before and after surgery. The AHI decreased from 52.69 ± 25.94 times/h to 26.98 ± 22.95 times/h, LsaO_2_ increased from 71.78 ± 11.87% to 78.70 ± 11.58%, ALT decreased from 44.29 ± 20.61 U/L to 26.47 ± 9.91 U/L, AST decreased from 27.71 ± 8.32 U/L to 21.82 ± 4.81 U/L, and UA decreased from 397.35 ± 92.14 umol/L to 362.94 ± 106.76 umol/L (*p* < 0.05).

### 3.3. Analysis of Factors Influencing the Changes in Liver- and Kidney-Function-Related Indexes in Patients with OSAHS after Minimally Invasive Surgery

As shown in Table 4, a correlation analysis of the factors influencing the improvement in liver- and kidney-function-related indicators in patients with OSAHS revealed correlations between ALT and LsaO_2_ (*r* = −0.635, *p* < 0.01), AST and LsaO_2_ (*r* = −0.504, *p* < 0.05), and UA and AHI (*r* = −0.532,*p* < 0.05).

## 4. Discussion

Previous studies have found that even if patients with OSAHS did not have obesity or metabolic syndrome, the prevalence of NAFLD would still increase significantly, and the severity of NAFLD was related to the increase in the severity of OSA [13]. In addition, more and more literature has begun to pay attention to the relationship between OSAHS and chronic kidney disease (CKD). They believed that OSAHS patients were 10 times more likely to have CKD disease than ordinary people, and that there may be a potential relationship between OSAHS and CKD. A two-way relationship, that was, OSAHS may increase the risk of kidney damage, while CKD may increase the risk of OSAHS. It may be that the mutual influence of the two parties cannot be determined due to the interaction of the two parties [14, 15]. In recent years, our team has used minimally invasive surgery to treat OSAHS and achieved good results [10–12]. But there have been few reports relating to the changes in the liver and kidney function indexes in patients with OSAHS after minimally invasive surgery. This study explored the significance of minimally invasive surgery on the changes of liver and kidney indexes in OSAHS patients by comparing the liver and kidney functions of 17 patients who met the 1-year follow-up.

The levels of ALT and AST in the OSAHS group were higher than in the control group, which is consistent with previous studies. Atan Doğan's [8] study of 194 patients with OSAHS and 114 control subjects found that the ALT and AST levels also in patients with OSAHS were higher than in the control group (*p* < 0.05). Qu Yinfei [9] found that the ALT, AST, and AHI levels of patients with OSAHS were positively correlated (*r* = 0.293, *p* < 0.05 and *r* = 0.275, *p* < 0.05), and ALT levels were negatively correlated with LSaO_2_ (*r* = −0.240, *p* < 0.05), suggesting that as the severity of OSAHS increases, ALT and AST levels increase. ALT and AST are the most sensitive test indicators of liver function damage. Many studies have shown that the severity of OSAHS is a risk factor for increased serum aminotransferase [10]. Patients with OSAHS have a risk of liver function damage. The reason for this may be that OSAHS causes repeated hypoxemia at night and increases oxidative stress, which causes damage to the liver cells.

In this study, the OSAHS group had higher levels of UA than the control group. Increased blood UA is primarily seen in patients with gout, and it is closely related to renal dysfunction. Studies have shown that with the continuous progression of chronic kidney injury, the glomerular filtration rate gradually decreases, and the ability of the kidney to excrete metabolic waste also decreases, which is manifested by rising continuous blood UA levels. UA is an index that is used clinically to assess the impairment of renal function. Asymptomatic patients with hyperuricemia are 1.6 times more likely to have chronic kidney injury than the general population [11]. This study also found that in the OSAHS group, UA was correlated with AHI and LsaO_2_ (*r* = 0.647 and -0.508, respectively; *p* < 0.01), suggesting that patients with OSAHS are at risk of renal impairment.

After minimally invasive surgery in patients with OSAHS, AHI decreased and LsaO_2_ increased, which objectively indicated that minimally invasive surgery improved the nighttime breathlessness and hypoxia of the patients, reduced the number of nighttime awakenings, and improved sleep quality. These findings are consistent with previous studies [10, 12], and the effectiveness of minimally invasive surgery has been confirmed by many studies.

There is no report of improvement in liver- and kidney-function-related indexes in patients with OSAHS who receive minimally invasive surgery. This study found that after minimally invasive surgery, the ALT levels decreased from 44.29 ± 20.61 to 26.47 ± 9.91 and the AST levels decreased from 27.71 ± 8.32 to 21.82 ± 4.81. Using correlation analysis, these decreases were found to be statistically significantly correlated with the change in LsaO_2_. It shows that minimally invasive surgery can reduce serum transaminases in patients with OSAHS, which may be related to the reduction of postoperative dyspnea, improvement of nocturnal hypoxia, and reduction of oxidative stress.

This study also found that after minimally invasive surgery, the UA levels in patients with OSAHS decreased, but no changes in urea or creatinine levels were found. There were no significant differences in the urea and creatinine levels between the OSAHS and the control group. Urea and creatinine are common indicators used in the clinical assessment of renal impairment, but the results are affected by many factors, such as age, gender, and muscle metabolism, and abnormalities often appear in the late stage of renal injury. However, the reduction in UA in patients with OSAHS after minimally invasive surgery was statistically significant, suggesting that minimally invasive surgery can improve uric acid metabolism in OSAHS patients.

However, our study still has limitations. The sample size we included is small, and the follow-up time is only 1 year. We will continue to expand the sample size and extend the follow-up time in future studies, looking forward to more accurate data.

## 5. Conclusion

In summary, this study showed that there are certain abnormalities in the levels of liver and kidney function indicators in patients with OSAHS, and minimally invasive surgery treatment can help to improve liver and kidney function damage in these patients. The research conclusions will be further confirmed by future follow ups [11, 12, 16, 17].

## Figures and Tables

**Table 1:** Comparison of basic information between the OSAHS group and the control group.

	OSAHS group	Control group	Value	*P*
Age	38.88 ± 9.48	35.94 ± 8.87	1.800^b^	0.074
Gender			0.246^a^	0.123
Male	48	68		
Female	3	11		
Occupation			1.000^a^	0.531
Mental work	26	41		
Manual work	25	38		
Smoking history			0.721^a^	0.423
Exist	23	33		
No	28	46		
Drinking history			0.594^a^	0.355
Exist	29	41		
No	22	38		

*Note.* “a” represents the chi-square value, “b” represents the *t* value; OSAHS, obstructive sleep apnea–hypopnea syndrome.

**Table 2:** Comparison of hematological indexes between the OSAHS group and the control group.

	OSAHS group	Control group	*t*	*P*
ALT (U/L)	48.98 ± 36.34	21.91 ± 11.61	6.154	<0.001
AST (U/L)	28.88 ± 14.80	22.18 ± 6.19	3.549	0.001
BUN (mmol/L)	4.66 ± 1.16	5.01 ± 1.31	-1.532	0.128
CREA (umol/L)	75.30 ± 11.00	76.59 ± 15.87	-0.504	0.615
UA (umol/L)	422.30 ± 98.65	330.49 ± 64.45	6.373	<0.001

OSAHS, obstructive sleep apnea–hypopnea syndrome; ALT, alanine aminotransferase; AST, aspartate transaminase; BUN, blood urea nitrogen; CREA, creatinine; UA, urea

**Table 3:** Changes in clinical indicators of OSAHS patients before and after minimally invasive surgery.

	Before surgery	After surgery	*t*	*P*
AHI	52.69 ± 25.94	26.98 ± 22.95	6.687	<0.001
LsaO_2_ (%)	71.78 ± 11.87	78.70 ± 11.58	-4.053	<0.001
MAI (times/h)	53.02 ± 20.26	47.70 ± 15.78	1.752	0.092
ALT (U/L)	44.29 ± 20.61	26.47 ± 9.91	4.395	<0.001
AST (U/L)	27.71 ± 8.32	21.82 ± 4.81	3.673	0.002
BUN (mmol/L)	4.92 ± 1.08	4.91 ± 0.79	0.032	0.975
CREA (umol/L)	72.81 ± 9.58	76.00 ± 10.33	-1.740	0.101
UA (umol/L)	397.35 ± 92.14	362.94 ± 106.76	2.580	0.020

*Note.* OSAHS, obstructive sleep apnea–hypopnea syndrome; AHI, apnea–hypopnea index; MAI, micro-arousal index; ALT, alanine aminotransferase; AST, aspartate transaminase; BUN, blood urea nitrogen; CREA, creatinine; UA, urea.

**Table 4:** Analysis of influencing factors of liver and kidney function related indexes of patients after minimally invasive surgery.

	AHI	LsaO_2_	MAI
ALT	0.413	-0.635^*∗∗*^	0.108
AST	0.357	-0.504^*∗*^	0.088
BUN	-0.199	0.288	0.192
CREA	-0.411	0.064	0.102
UA	-0.523^*∗*^	0.016	0.093

*Note.* ^*∗*^ *p* < 0.05, ^*∗∗*^*p* < 0.01; AHI, apnea–hypopnea index; MAI, micro-arousal index; ALT, alanine aminotransferase; AST, aspartate transaminase; BUN, blood urea nitrogen; CREA, creatinine; UA, urea

## Data Availability

The datasets used and/or analysed during the current study available from the corresponding author on reasonable request.

## Abbreviations

(OSAHS): Obstructive sleep apnea–hypopnea syndrome
(NAFLD): Nonalcoholic fatty liver disease
(PSG): Polysomnography
(AHI): Apnea–hypopnea index
(ALT): Alanine aminotransferase
(AST): Aspartate aminotransferase
(UA): Uric acid
(CKD): Chronic kidney disease.
